# GC-MS and HPLC chemical profile, antioxidant, anti-acetylcholinesterase, and anti-diabetic activities of Libyan *Salvia lanigera* herb extract and essential oil

**DOI:** 10.1038/s41598-025-12233-x

**Published:** 2025-08-29

**Authors:** Fatma A. Elshibani, Amr Farouk, Esra El Naili, Engy Mahmoud, Ahmed Othman, Hamdoon A. Mohammed, Nagla Elkazza, Mohamed A. Sharkasi, Nada A. Alzunaidy

**Affiliations:** 1 Department of Pharmacognosy, Faculty of Pharmacy, University of Benghazi, Benghazi, Libya; 2 Flavour and Aroma Chemistry Department, National Research Centre, Dokki, Giza, 12622 Egypt; 3 Department of Pharmaceutical Chemistry, Faculty of Pharmacy, University of Benghazi, Benghazi, Libya; 4 Department of Pharmacognosy and Medicinal Plants, Faculty of Pharmacy, Al-Azhar University, Cairo, Egypt; 5 Department of Medicinal Chemistry and Pharmacognosy, College of Pharmacy, Qassim University, Buraidah, 52571 Saudi Arabia; 6 Department of Pharmacology, Faculty of Pharmacy, Assalam International University, Benghazi, Libya; 7 Department of Food Science and Human Nutrition, College of Agriculture and Food, Qassim University, Buraydah, 51452 Saudi Arabia

**Keywords:** Anti-cholinesterase activity, Antidiabetic effects, Flavonoids, Antioxidants, HPLC-DAD, Phenolic acids, *Salvia lanigera*, Biochemistry, Chemical biology, Drug discovery

## Abstract

The current study explored the chemical composition and biological activities of the essential oil and ethanol extract of S*alvia lanigera* collected from Libya. The ethanol extract obtained from the wild growing *S*. *lanigera* was evaluated for the chemical composition via high-performance liquid chromatography with diode-array detection (HPLC–DAD), which demonstrated phenolic and flavonoid contents. A total of 17 compounds, representing phenolic and flavonoid derivatives, have been identified. Besides, gas chromatography–mass spectrometry (GC–MS) analysis of the essential oil obtained from S. *lanigera* aerial parts identified 24 compounds, accounting for 99.33% of the oil, and the major components were characterized to be 1,8-cineole (27.28%), camphor (25.82%), α-pinene (7.71%), and α-terpineol (7.67%). The results indicated that the oil and ethanol extract exhibited marked radical scavenging capacity toward DPPH (2,2-diphenyl-1-picrylhydrazyl), with IC_50_ values of 0.1337 and 0.6331 µg/mL, respectively. Further, the oil and ethanol extract demonstrated potent activity against ABTS (2,2′-azino-bis(3-ethylbenzothiazoline-6-sulfonic acid)), with IC_50_ values of 0.17 and 0.0501 µg/mL, respectively. Moreover, *S. lanigera* oil inhibited more than 76% of the acetylcholinesterase enzyme activity, with an IC_50_ value of 144 ± 1.04 µg/mL. The in vitro data showed that the ethanol extract has potential antidiabetic properties toward the *α*-glucosidase enzyme with an IC_50_ value of 124.6 ± 1.07 µg/mL. Regarding the nature of measurement, assay conditions, and ligand-specific factors such as solubility and stability, we demonstrated a correlation between the IC_50_ values and in-silico results for antidiabetic assays, where chlorogenic and rosmarinic acids recorded the highest docking scores of -9.4 and − 8.9 kcal/mol, respectively. However, this correlation was not observed in the anti-cholinesterase assay. Further in vivo investigations are essential to confirm the in vitro results of *S. lanigera* oil and ethanolic extract. This study helps elucidate the potential therapeutic applications of these compounds and their effectiveness in managing diabetes and neurodegenerative disorders.

**Supplementary Information:**

The online version contains supplementary material available at 10.1038/s41598-025-12233-x.

## Introduction

The genus *Salvia* L., commonly known as Sage, is the largest genus in the Lamiaceae family, comprising over 1000 perennial and annual species distributed worldwide. The genus *Salvia* is represented in Libyan flora with ten species, including *S. lanigera*,* S. fruticosa*, *S. aegyptiaca*, *S. verbenaca*, *S. chudaei*, *S. spinosa*, *S. viridis*, *S. officinals*, *S. coccinea*, and *S. splendens*. Across the world, many researchers are interested in investigating the potential of *Salvia* species, attributed to its economic and therapeutic importance, as several Sage species have been traditionally utilized for various ailments, including diarrhoea, gonorrhoea, respiratory problems, wounds, eye diseases, diabetes, inflammation, and impaired cognition. Thus, various studies have documented the biological properties of the essential oils extracted from *Salvia* species with antibacterial and antiviral activities^1,2^. Several species of the genus *Salvia* exhibited antifungal and antibacterial properties toward various food microorganisms. Hence, they are used as natural food preservatives^3^. Further, bioactive secondary metabolites have been discovered in plants of this genus, including terpenes, diterpene quinones, phenolics, and their derivatives^4,5^. Among *Salvia* plants, *S. lanigera* is a small perennial herb characterized by violet inflorescences, which inhabits low-altitude deserts and sandy loams. *Salvia lanigera* is well-recognized by its deep violet color of corolla and covered with short-erect hairs giving it its name “Lanigera”, which means “fleecy” or “wool-bearing”. *Salvia lanigera* has been reported in North Africa, including Egypt and Libya, Turkey and Iran^6^. It has been reported that *S. lanigera* is commonly used by Bedouins of the Sinai Peninsula as a condiment for tea^7^. Previous studies conducted on the essential oil (EO) from *S. lanigera* have revealed antimicrobial, antioxidant, and antitumor properties^3,6^.

Alzheimer’s disease (AD) is a brain neurodegenerative disease associated with the deficiency of acetylcholine, a neurotransmitter with crucial role in learning and cognition. This disease affects brain regions, leading to impaired thinking and memory. Currently, one of the most important medications for the management of AD is cholinesterase inhibitors^8,9^.

Diabetes mellitus (DM) is a disease characterized by hyperglycaemia, and nowadays, it is considered a fatal metabolic disorder with severe complications, including retinopathy, nephropathy, peripheral neuropathy, arterial disease, and infertility. By 2050, about 600 million people will have diabetes, which raises serious concerns on health care systems^10^. Nowadays, the two most common health issues are AD and DM. It has been noted that DM increases the risk of dementia and cognitive decline, particularly AD and vascular dementia^11^. Accordingly, there is an urgent need to discover natural-derived products for managing AD and DM. In that context, huge research has been conducted to evacuate the beneficial effects of medicinal plants and their product in treating both AD and DM^12,13^. Importantly, previous studies have elucidated *Salvia* herbs’ bioactive compounds and pharmacological potential, which were collected from different geographical regions, including the Sinai Peninsula, Egypt, and Libya^14^. Furthermore, the observed beneficial biological properties were attributed to several bioactive compounds, including monoterpenes, oxygenated monoterpenoids, sesquiterpenes, phenolic acids, flavonoids, and phenylpropanoids. To the best of our knowledge, limited studies have been conducted on *S. lanigera* growing Libya. Therefore, this study aimed to evaluate the chemical composition of ethanol extract and EO of the aerial parts of *S. lanigera* herb, as well as to evaluate the in vitro antioxidant, neuroprotective, and antidiabetic properties. Consequently, the study focused on *S. lanigera* EOs, phenolic and flavonoid contents that may exhibit strong biological properties and potentially be utilized as therapeutic agents and food preservatives.

## Materials and methods

### Chemicals and plant material

Gallic acid, ABTS solution, and DPPH (≥ 90%) were sourced from Merck KGaA (Darmstadt, Germany), while all other chemicals and reagents were purchased from Sigma-Aldrich (St. Louis, MO, USA). The aerial parts of the plant were collected from the Eastern area of Libya during the spring season of 2023. The taxonomist, Dr. Salem Elshatshat, identified the plant as *S. lanigera* and and a voucher specimen was deposited under the accession number PSL-2406 at herbarium of the Pharmacognosy and Natural Products Laboratory, Faculty of Pharmacy, Assalam University, Libya.

### Sample preparation

#### Extract preparation

The dried aerial parts of *S. lanigera* (200 g) were extracted three times by maceration with 95% ethanol. The combined ethanol extracts were dried under reduced pressure.

#### Essential oil extraction

Using the Clevenger apparatus, the fresh aerial parts (500 g) were hydrodistilled for three hours at 75 °C. The oil was then collected, dried over anhydrous sodium sulphate (Na_2_SO_4_), and stored in amber glass containers at 4 °C.

### Gas chromatography-mass spectrometry (GC-MS) analysis

The oil volatiles were separated using a gas chromatograph (Agilent 8890 System, Delaware, USA) coupled with a mass spectrometer (Agilent 5977B GC/MSD) that had an HP-5MS capillary column (30 m, 0.25 mm i.d., 0.25 mm film thickness). A sample size of 1 µL was injected at 230 °C in split mode (1:50). The oven temperature was initially set at 50 °C and then increased at a rate of 5 °C/min to 200 °C. It was subsequently raised to 280 °C at 10 °C/min, where the temperature was maintained isothermally for 7 min. Mass spectra were acquired in electron impact (EI) mode at 70 eV, covering a mass-to-charge ratio (*m*/*z*) range from 39 to 500 amu. Peaks were identified by comparing them to NIST standards and published data. The percentages of detected compounds were calculated based on the GC peak areas. The Kovats index for each compound was determined using the retention times of C6–C26 *n*-alkanes (Supelco Inc., Bellefonte, PA, USA) and compared to values found in the literature^15^.

### Determination of phenolics using High-Performance liquid chromatography (HPLC)

HPLC analysis was carried out using an Agilent 1260 series. The separation was carried out using Zorbax Eclipse Plus C8 column (4.6 mm x 250 mm i.d., 5 μm). The mobile phase consisted of water (A) and 0.05% trifluoroacetic acid in acetonitrile (B) at a flow rate of 0.9 mL/min. The mobile phase was programmed consecutively in a linear gradient as follows: 0 min (82% A); 0–1 min (82% A); 1–11 min (75% A); 11–18 min (60% A); 18–22 min (82% A); 22–24 min (82% A). The multi-wavelength detector was monitored at 280 nm. The injection volume was 5 µl for each of the sample solutions. The column temperature was maintained at 40 °C^16^.

### Antioxidant activity measurements

#### DPPH radical scavenging assay

A spectrophotometric method using 2,2-diphenyl-1-picrylhydrazyl (DPPH) as a reagent was used to determine the radical scavenging activity of the extract and essential oil obtained from *S. lanigera*. TROLOX and ascorbic acid was serve as reference standards. The total reaction volume was 3 mL of a methanol solution containing DPPH radicals. The mixture was vigorously shaken and kept in the dark for 30 min. At 517 nm, the absorbance of the mixture was measured thrice for accuracy using a Shimadzu UV-160-IPC spectrophotometer (Kyoto, Japan) against a blank. The result was calculated using the following formula: I%=[(ΔA517C-ΔA517S)/ΔA517S], where ΔA is the average absorbance, C is the control, and S is the sample^17^.

#### The ABTS free radical scavenging assay

The second test to evaluate antioxidant activity was the 2,2′-Azino-bis (3-ethylbenzothiazoline-6-sulfonic acid) di-ammonium salt radical cation (ABTS+). A Shimadzu spectrophotometer UV-160-IPC (Kyoto, Japan) measured the absorbance at 734 nm to determine reducing power. Ascorbic acid was used as a positive control, whereas deionized water was used as a blank^17^.

### Quantitative analysis of phenolics and flavonoids

#### Total phenolic content

Folin-Ciocalteu reagent and gallic acid were used to determine phenolic content. The reaction mixture was incubated at 45 °C for 45 min and measured at 765 nm^3^. A 100 µL of the sample was mixed with 1.5 mL of the Folin’s phenol reagent (10%). After 5 min, 0.5 mL of saturated sodium carbonate (Na_2_CO_3_) (7.5%) was added. The absorbance of the supernatant was measured at 725 nm. TPC was standardized against gallic acid (GA) and expressed in terms of mg GA equivalents (GAE)/mL of dry matter (DM). This assay’s linearity range was determined as 0.02–0.3 mg GA/mL (R2 = 0.99). The analysis was repeated in triplicate.

#### Estimation of flavonoid content

Using a calibration curve based on rutin, the flavonoid content in the ethanol extract has been measured. A 100 µL of the sample was mixed with 0.1 mL of sodium nitrite (NaNO_2_) and 0.1 mL of aluminium chloride (AlCl_3_) solution, and the mixture incubated for 5 min. Then, 2 mL of 4% sodium hydroxide (NaOH) was added, followed by incubation for 10 min. Finally, the absorbance at 510 nm was measured to determine the flavonoid content expressed as milligrams per gram of rutin equivalent^17^.

### Enzyme inhibition activity

#### α-Amylase inhibition assay

The assay followed the method of Khadayat et al.^18^. In 96-microwell plates, 20 µL of samples or blanks were combined with 140 µL phosphate buffer (50 mM, 0.9% NaCl, pH 7). Then, 20 µL of amylase enzyme (1 mg/mL) was added, and the mixture was incubated for 15 min at 37 °C. Next, 20 µL of substrate (0.375 mM) was added, followed by an additional 10-minute incubation at 37 °C. Enzyme activity was measured by releasing p-nitrophenol at 405 nm using a microplate reader (Onega, USA). The percentage of α-amylase inhibition was calculated using the formula:

% Inhibition = [(A blank – A sample)/A blank] x 100.

Here, A blank is the absorbance of the control (without inhibitor), and A sample is the absorbance in the presence of the inhibitor.

#### α-Glucosidase inhibition assay

The assay followed the method of Abdallah et al.^19^. In 96-microwell plates, 25 µL of samples or blanks were incubated for 10 min at 37 °C with 50 µL of α-glucosidase from *Saccharomyces cerevisiae* (0.6 U/mL) in phosphate buffer (0.1 M, pH 7). Subsequently, 25 µL of 3 mM *p*-NPG substrate was added, and the mixture was incubated for an additional 5 min at 37 °C. Enzyme activity was measured by releasing *p*-nitrophenol at 405 nm using a microplate reader. The percentage of α-glucosidase inhibition was calculated as follows:

% Inhibition = [(A blank – A sample)/A blank] x 100.

A blank is the control absorbance, and A sample is the absorbance with the inhibitor.

#### Acetylcholine esterase inhibition assay

The assay followed the Elmann et al.^20^ and Osman et al.^21^ methods, with minor modifications. In a 96-well plate, 10 µL of indicator solution (0.4 mM in 100 mM tris buffer pH 7.5) was added, followed by 20 µL of enzyme solution (0.02 U/mL acetylcholine esterase in 50 mM tris buffer pH 7.5 with 0.1% BSA) and 20 µL of sample/standard solution. After adding 140 µL of buffer, the mixture was incubated for 15 min at room temperature. Then, 10µL of acetylcholine iodide substrate was added, and the plate was incubated for 20 min in the dark. The color was measured at 412 nm, and data are presented as means ± SD.

### Docking study

Enzyme crystal structures were downloaded from the Protein Data Bank (PDB) (https://www.rcsb.org/) on April 24, 2025, including human pancreatic α-amylase (PDB ID: 4GQR), α-glucosidase (PDB ID: 3A4A), and human acetylcholinesterase (PDB ID: 4EY7). Water and ligand molecules were removed and protonated using PyMOL (version 2.5.1). Major phytochemicals as ligands were obtained from PubChem via http://pubchem.ncbi.nlm.nih.gov/ on the same date and optimized with Avogadro (version 1.2.0). The binding potential of the enzyme’s pockets was predicted using CB-DOCK2 and Discovery Studio (ver25.1.0.24284). The docking method was validated by re-docking co-crystallized ligands (**Supplementary file**) with AutoDock 1.5.6 and Vina^22^yielding low RMSD values between 0.547 and 0.941 Å. Based on the identified pocket residues and dimensions, docking was performed using AutoDock Vina through CB-DOCK2 (http://clab.labshare.cn/cb-dock/php/) on April 24–25, 2025^23^. The docked complexes were analyzed and visualized with Discovery Studio^24^.

## Results and discussion

### Phytochemical analysis of *Salvia lanigera* extract and essential oil

The current study investigated the chemical profile of *S. lanigera* extract and essential oil (EO). In the study, the aerial parts of *S. lanigera* were extracted via maceration with ethanol (95%) and hydrodistilled by the Clevenger apparatus. Further, the extract was phytochemically analyzed using HPLC-DAD against specific phenolics and flavonoids standards. Additionally, the distilled EO was phytochemically profiled using gas chromatography-mass spectrometry (GC-MS) analysis. The family Lamiaceae and *Salvia* species are aromatic plants known for their production of essential oils^25,26^. These plants are a valuable source of biologically active phenolic acids and flavonoids^26^. However, the plant production of the essential oils, phenolic acids, and flavonoids is highly affected by several abiotic and biotic factors. These factors are also affecting the plants’ biological activities and therapeutic indications^27^.

**Fig. 1:**
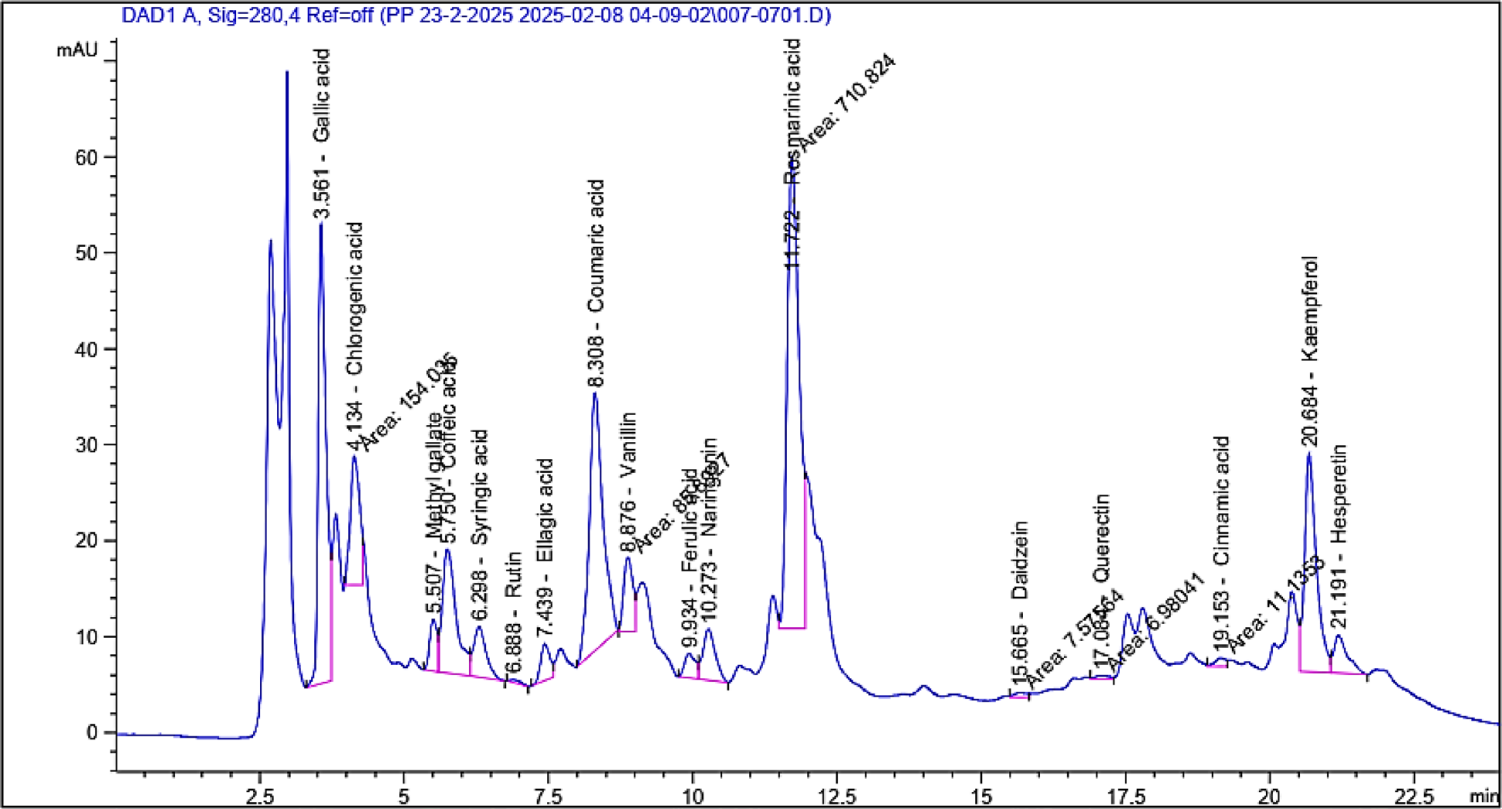
HPLC chromatogram of *S. lanigera* ethanol extract.

**Fig. 2:**
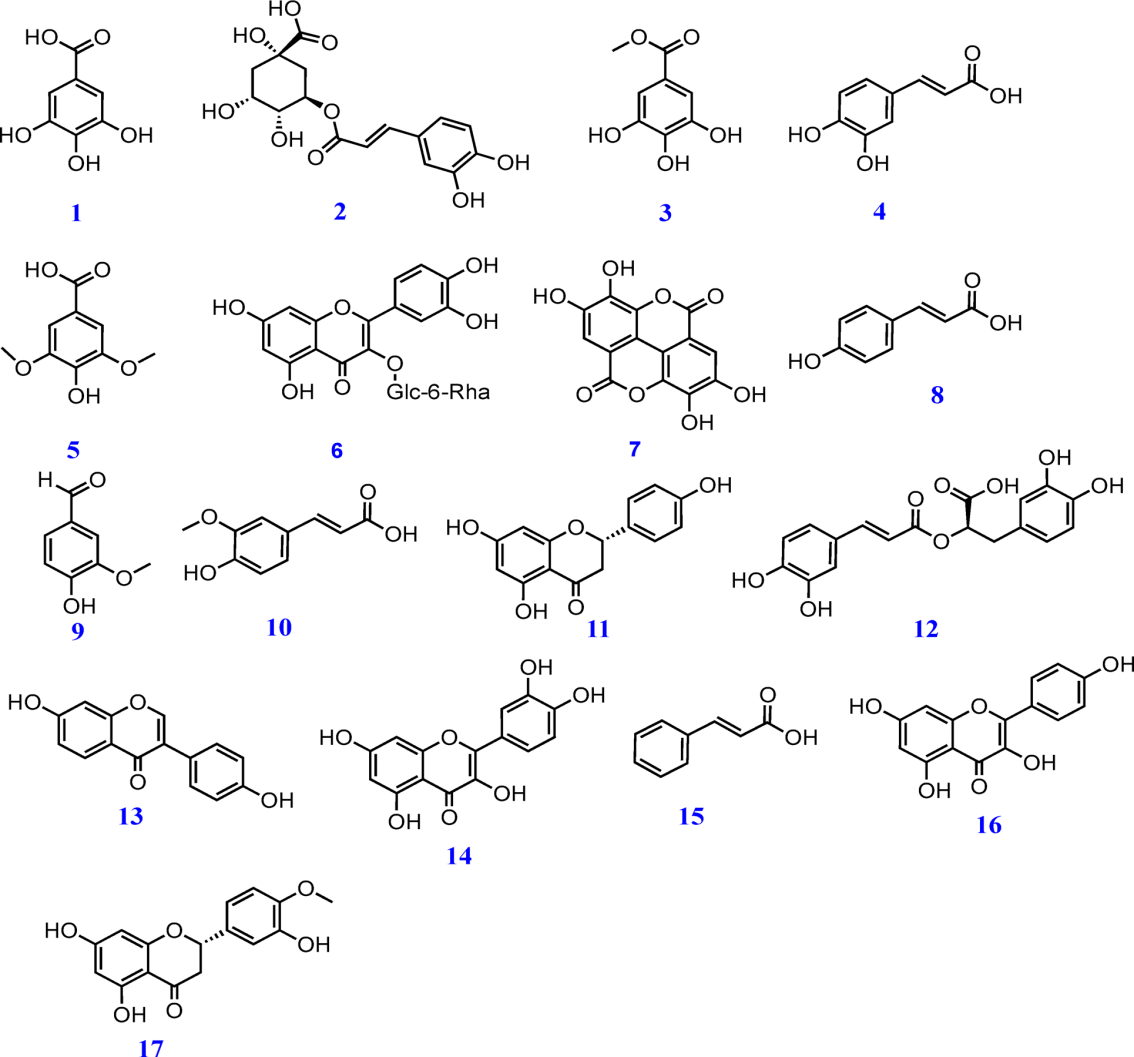
Chemical structures of the identified chemical compounds: gallic acid (**1**), chlorogenic acid (**2**), methyl gallate (**3**), caffeic acid (**4**), syringic acid (**5**), rutin (**6**), ellagic acid (**7**), coumaric acid (**8**), vanillin (**9**), ferulic acid (**10**), naringenin (**11**), rosmarinic acid (**12**), daidzein (**13**), quercetin (**14**), cinnamic acid (**15**), kaempferol (**16**), and hesperetin (**17**).

In HPLC analysis, the chemical compounds were determined based on comparing their retention times with those of reference standards, and they were characetrized as gallic acid, chlorogenic acid, catechin, methyl gallate, caffeic acid, syringic acid, ellagic acid, rutin, coumaric acid, vanillin, ferulic acid, naringenin, rosmarinic acid, daidzein, quercetin, cinnamic acid, kaempferol, and hesperitin as shown in Table 1 and Figs. 1 and 2. Notably, marked higher contents of phenolic acids, totally calculated as 8387.68 µg/g of the plant extract, were revealed via HPLC analysis. Moreover, phenolic acid derivatives, including gallic acid and chlorogenic acid, were determined at contents more than 1000 µg/g (1675.66 and 1073.21 µg/g of the plant extract, respectively. Additionally, the study outcomes revealed the presence of coumaric acid, caffeic acid, ellagic acid, syringic acid, ferulic acid, vanillin, and rosmarinic acid in considerable amounts of 798.00, 553.82, 213.39, 234.05, 96.61, 155.76, and 3451.75 µg/g of the plant extract, respectively. Flavonoids, including kaempferol, naringenin, and hesperetin were determined at contents of 406.91, 371.34, and 152.61 µg/g, respectively. The findings in Table 1 and Fig. 1 are consistent with the reported phenolic acids and flavonoids of plants grown in various regions, such as Egypt^1^ and also revealed variations in polyphenolic nature, which may reflect the effect of the growing environment on the plant.

**Table 1:** Phenolics and flavonoids identified in *Salvia lanigera* ethanol extract by HPLC analysis and their quantified contents.

No.	Compound	Area	Content (µg/mL)	Content (µg/g)
1	Gallic acid	457.72	33.51	1675.66
2	Chlorogenic acid	154.03	21.46	1073.21
3	Methyl gallate	48.41	2.71	135.44
4	Caffeic acid	215.90	11.08	553.82
5	Syringic acid	79.58	4.68	234.05
6	Rutin	3.92	0.59	29.35
7	Ellagic acid	42.01	4.27	213.39
8	Coumaric acid	444.08	15.96	798.00
9	Vanillin	85.89	3.12	155.76
10	Ferulic acid	33.29	1.93	96.61
11	Naringenin	80.47	7.43	371.34
12	Rosmarinic acid	710.82	69.03	3451.75
13	Daidzein	7.58	0.43	21.69
14	Querectin	6.98	0.87	43.47
15	Cinnamic acid	11.14	0.22	10.79
16	Kaempferol	323.72	8.14	406.91
17	Hesperetin	65.18	3.05	152.61

**Fig. 3:**
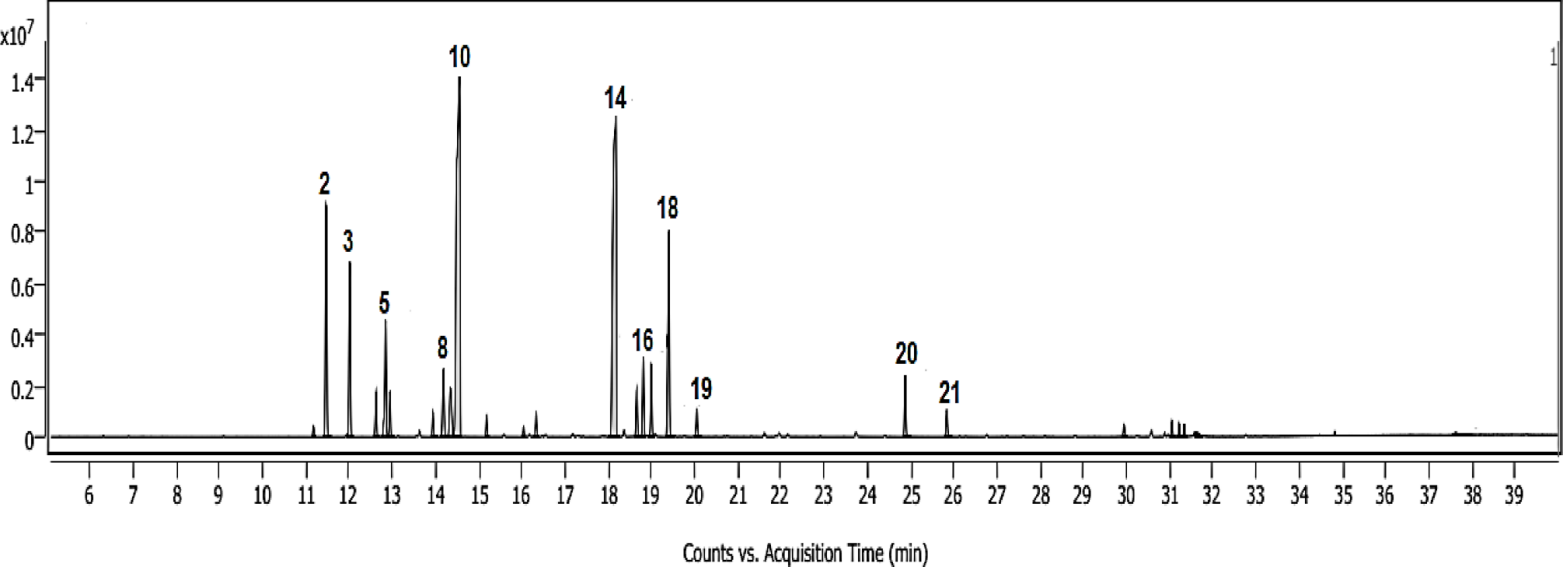
GC-MS Chromatogram of *S. lanigera* oil.

**Fig. 4:**
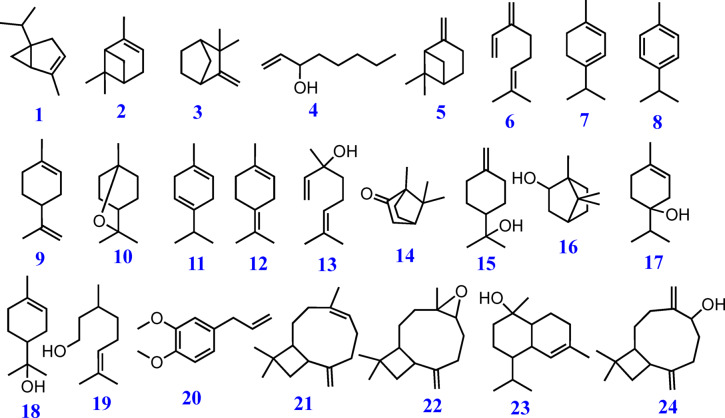
Structures of identified metabolites using GC-MS analysis.

The volatile constituents of *S. lanigera* were identified via GC-MS analysis (Figs. 3 and 4). The identification was confirmed by comparison with the retention indices, the mass spectrum of authentic compounds, and the NIST mass spectra library data. As shown in Table 2, GC–MS analysis was used to identify 24 volatile compounds in the *S. lanigera* sample, accounting for 99.33% of all compounds detected. The compounds **1**–**24** identified were categorized into different chemical classes, including monoterpene, sesquiterpene, phenylpropene, and fatty alcohol derivatives.

In the present study, monoterpenes derivatives were characterized, including oxygenated bicyclic hydrocarbon (1,8-cineole **10**, borneol **16**, camphor **14**, 55.6%), representing the major oil constituents, cyclic hydrocarbon (α-terpinene **7**, D-limonene **9**, γ-terpinene **11**, terpinolene **12**, 4.22%), oxygenated cyclic hydrocarbon (δ-terpineol **15**, terpinen-4-ol **17**, α-terpineol **18**, 11.34%), oxygenated acyclic hydrocarbon (linalool **13**, citronellol **19**, 1.56%), acyclic hydrocarbon (β-myrcene **6**, 1.27%), and bicyclic hydrocarbon (α-thujene **1**, α-pinene **2**, camphene **3**, β-pinene **5**, 17.47%). *p*-Cymene **8** (2.62%) was detected as an aromatic hydrocarbon, whereas methyl eugenol **20** (1.84%**)** was determined as phenylpropene. Moreover, 1-octen-3-ol **4** (1.39%) was determined as fatty alcohol derivative in the essential oil of *S. lanigera.* Additionally, four sesquiterpene compounds were determined, classified as bicyclic hydrocarbons, including caryophyllene **21**, caryophyllene oxide **22**, *tau*-cadinol **23**, and α-betulenol **24**, 1.92%.

The literature claimed that the chemical profile of EOs exhibits variability depending on the maturity stage of the plant, the time of harvest, geographical location, and prevailing environmental conditions^28^. Previous studies revealed that monoterpene derivatives were the main chemical class of the essential oil reported in *S. lanigera* with 71.7%, followed by sesquiterpenes with 21.7% and phenylpropanoid derivatives with (3.5%). The high content of monoterpenes was mainly due to the presence of thymol (54.9%). Other constituents included cedrol (8.9%), methyl chavicol (3.5%) and spathulenol (3.4%). The reported essential oil was mainly composed of oxygenated derivatives (85.9%), classified as alcohols, ketones, aldehydes and phenols^29^.

**Table 2:** Salvia lanigera oil composition using GC-MS analysis.

No.	Compound	RI^a^	LRI^b^	Area%	Identification^c^
1	α-Thujene	935	930	0.29	RI, MS
2	α-Pinene	942	939	7.71	RI, MS, STD
3	Camphene	958	954	5.51	RI, MS, STD
4	1-Octen-3-ol	983	979	1.39	RI, MS
5	β-Pinene	985	979	3.96	RI, MS, STD
6	*β*-Myrcene	992	990	1.27	RI, MS, STD
7	α-Terpinene	1021	1017	0.75	RI, MS
8	*p*-Cymene	1025	1024	2.62	RI, MS, STD
9	D-Limonene	1031	1029	2.65	RI, MS, STD
10	1,8-Cineole	1035	1031	27.28	RI, MS, STD
11	γ-Terpinene	1058	1059	0.62	RI, MS
12	Terpinolene	1092	1088	0.30	RI, MS
13	Linalool	1100	1096	0.74	RI, MS
14	Camphor	1150	1146	25.82	RI, MS, STD
15	δ-Terpineol	1169	1166	1.45	RI, MS
16	Borneol	1175	1169	2.50	RI, MS
17	Terpinen-4-ol	1181	1177	2.22	RI, MS
18	α-Terpineol	1191	1188	7.67	RI, MS, STD
19	Citronellol	1228	1225	0.82	RI, MS
20	Methyl eugenol	1405	1403	1.84	RI, MS
21	Caryophyllene	1421	1419	0.82	RI, MS
22	Caryophyllene oxide	1582	1583	0.37	RI, MS
23	*tau*-Cadinol	1641	1640	0.41	RI, MS
24	α-Betulenol	1669	1667	0.32	RI, MS
	**Total**	–	–	**99.33**	–

**RI**^**a**^: Retention indices were calculated using the DB-5 column using alkane standards. **LRI**^**b**^: Retention indices according to literature. ^**c**^ Confirmed by comparison with the retention indices, the mass spectrum of the authentic compounds, and the NIST mass spectra library data.

### Total phenolics and flavonoids

The phenolic and flavonoid contents of the plant EO and extract were also measured, and their quantities in equivalents to gallic and rutin are presented in Table 3. The findings indicated comparable results for the phenolic contents of both EO and the plant extract. The phenolic contents in EO are mainly related to the phenolic volatile oils, such as methyl eugenol, which was detected at a relative concentration of 1.84% (Table 1). However, phenolic contents of the extract are consistent with the presence of gallic acid, chlorogenic acid, and rosmarinic acid at higher concentrations, as demonstrated in Table 1. The flavonoid contents were only determined in the plant extract and revealed the presence of 65.08 rutin equivalent per g of the extract. The current findings for the plant’s phenolic and flavonoid contents do not agree with the literature, which measures *S. lanigera* phenolic and flavonoid contents at 43.04–67.39 mg GAE/g and 26.42–51.59 mg QE/g, respectively^6^. This could be due to a variety of factors, including the plant’s collection time and variations in the flavonoid standard used to determine total flavonoid content.

**Table 3:** Total phenolic and flavonoid contents, and antioxidant activities of *Salvia lanigera* essential oil and ethanol extract.

Samples	TPC*	TFC**	DPPH***	ABTS***
Essential oils	11.60 ± 0.12	nd	0.13	0.17
Extract	11.34 ± 0.41	65.08 ± 1.12	0.63	0.05

*Total phenolic contents calculated as gallic acid equivalents per gram of the oil or extract; **Total flavonoid contents calculated as rutin equivalents per gram of the oil or extract; ***IC_50_ against DPPH and ABTS free radicals calculated as µg/mL. nd, not determined.

### The antioxidant potential of Salvia lanigera oil and ethanol extract [DPPH and ABTS radical scavenging assay

This study delved into the antioxidant activity of *S. lanigera* EO and ethanol extract as radical scavenger potential against DPPH and ABTS free radicals. The study outcomes demonstrated that *S. lanigera* EO and ethanol extract had marked radical scavenging capacity in a dose-dependent manner. Importantly, *S. lanigera* oil and ethanol extract showed marked radical scavenging activity toward DPPH, with IC_50_ values of 0.1337 and 0.6331 µg/mL, respectively (Table 3). Similar scavenging capacity patterns were discovered in the ABTS assay. Notably, EO and ethanol extract demonstrated potent activity at a concentration of 150 µg/mL, with IC_50_ values of 0.17 and 0.0501 µg/mL, respectively. These findings highlight the potent antioxidant activity of *S. lanigera* EO and its ethanol extract. These results are inconsistent with the reported antioxidant activity of the plant^6,30,31^ and can be correlated to the presence of several antioxidant compounds in the plant EO, e.g., 1,8-cineole^32,33^. The presence of several well-known antioxidant phenolic acids, e.g., caffeic acid, gallic acid, rosmarinic acid, and coumaric acid, and flavonoids, e.g., rutin and quercetin, is also attributed to the antioxidant activity of the plant extract. This suggests that the overall antioxidant capacity may be influenced not only by the individual compounds but also by their synergistic interactions.

### Evaluation of the anticholinesterase potential of *Salvia lanigera* extract and essential oil

Acetylcholine (ACh) is a brain neurotransmitter that has an important role in managing Alzheimer’s disease (AD). It plays a crucial role in learning and memory development. It is important to note that cholinesterase inhibitors are medications most frequently recommended to manage AD. Traditionally, plants of the genus *Salvia* are well-recognized for their memory-enhancing properties^34^. Thus, the current study tested the inhibition capacity of *S. lanigera* EO toward acetylcholinesterase enzyme (AChE). At a concentration of 500 µg/mL, *S. lanigera* EO inhibited more than 76% of AChE activity with an IC_50_ value of 144 ± 1.04 µg/mL as seen in Table 4. These findings are consistent with previous reports in the literature, which revealed a significant activity of various *Salvia* species, particularly rosmarinic acid, which showed neuroprotective properties^34^.

*Salvia* species have been associated with neuroprotective properties. Importantly, the lipophilicity of EO components making them capable of passing the blood–brain barrier and thus could be utilized as a significant strategy for the treatment of neurodegenerative disorders^35^. EOs are widely applied in food, hair, and skin treatments, indicating that they are generally well tolerated. Compared to synthetic drugs, EOs contain a variety of compounds that perform their action via synergism to reduce the risk of drug resistance and improve the efficacy of treatment^36^.

It is worth mentioning that the EO composition of *S. lanigera* growing in Libya has not been fully investigated yet. To the best of our information, the investigation of inhibitory activity of *S. lanigera* EO against acetylcholinesterase enzyme is reported here for the first time and those of *S. sharifii*, *S. santolinifolia*, *S. reuterana*, *S. spinosa*, *S. palaestina*, *S. virgata*, *S. hypoleuca*, *S. mirzayanii*, *S. sclarea*, *S. verticillata*, *S. multicaulis*, and *S. syriaca* EOs were previously evaluated^37^. Besides, literature survey indicated that the AChE inhibition activity of ethanol and water extracts of *S. fruticosa* and *S. lanigera* at concentrations 25–100 µg/mL was evaluated. The ethanol extract of *S. lanigera* (100 µg/mL) exhibited a weaker inhibition activity (31.03 ± 0.43%) than positive control, galanthamine (57.11%)^6^.

Previous studies have discovered that α-pinene, β-pinene, 1,8-cineole, camphor, borneol, α-thujone and β-thujone, thymol, caryophyllene, and caryophyllene oxide have been characterized as predominant components in EOs obtained from *Salvia* species. Besides, caryophyllene oxide was reported as the most abundant compound in all EOs of various *Salvia* plants^37^.

Previous investigation of the acetylcholinesterase inhibition activity EO obtained from *S. verticillata* demonstrated a weak effect with 20.4% inhibition, which was consistent with results reported by Gharehbagh and her team^37^. Additionally, *S. syriaca* EO exhibited a potent AChE inhibition activity with an IC_50_ value of 1.90 ± 0.1 mg/mL, as compared to the positive drug, galantamine (IC_50_ = 2.59 ± 0.01 mg/mL). Conversely, Gharehbagh and her team reported that *S. syriaca* EO (500 µg/mL) demonstrated a weak AChE inhibition activity with an IC_50_ value of 15.8 ± 0.5%, as compared to donepezil (IC_50_ = 89.9 ± 0.12)^37^.

The presence of α-pinene, 1,8-cineole, linalool, limonene, and myrtenyl acetate in the EO of *Myrtus communis* leaves have been correlated with anti-cholinesterase, antioxidant and neuroprotective effects^38^. Further, α-pinene and 1,8-cineole of *S. leriifolia* exerted promising BChE inhibition activities with IC_50_ values of 0.87 and 0.93 mM, respectively^39^. Additionally, α-pinene showed a marked inhibition activity against AChE with 76.3 ± 1.27% inhibition^40^. Caryophyllene oxide from *S. verticillate* showed a strong inhibition effect against AChE and BuChE (61.03 ± 3.81% and 41.46 ± 2.66%, respectively)^41^. Noteworthy, 1,8-cineole, a monoterpenoid compound reported in many plant EOs has been shown to possess anticholinesterase activity and beneficial neuroprotective properties, as it could modulates tau phosphorylation through down-regulating the activity of GSK-3β and inhibiting the BACE1 activity and consequently reducing Aβ production. Hence, 1,8-cineole was suggested as a therapeutic agent in the treatment of AD^42,43^. Furthermore, the acetylcholinesterase inhibitory activity for the *Cinnamomum camphora* EO at concentration of 1 mg/mL revealed 53.61 ± 2.66% inhibition, as compared to standard drug physostigmine 97.53 ± 0.63% at 100 ng/mL. Apparently, the EO detected in *C. camphora* was found rich in 1,8-cineole (55.84%), sabinene (14.37%), and α-terpineol (10.49%), that might contribute to AChE inhibition activity^44^. Interestingly, 1,8-cineole and α-terpineol were characterized here in *S. lanigera* EO with 27.28 and 7.67%, respectively.

In the present study, GC–MS analysis of *S. lanigera* EO revealed that monoterpenes, phenyl propene and sesquiterpenes were determined in EO from *S. lanigera*. Among them, 1,8-cineole, camphor, α-pinene, α-terpineol, caryophyllene oxide and other sesquiterpenes have been determined. In this work, the EO of *S. lanigera* growing in Libya, exerted strongest activity toward AChE. In this regard, it seems that α-pinene, 1,8-Cineole, α-terpineol, linalool, limonene, sesquiterpenes, and other volatile constituents detected in EO of *S. lanigera* contribute to the acetylcholinesterase inhibition properties. Thus, *S. lanigera* might be a potential and safe source for new medicinal agents to control AD.

**Table 4:** Anti-Acetylcholinesterase and Anti-α-Glucosidase activities (IC₅₀ values, µg/mL) of *Salvia lanigera* (SL) extract and essential oil.

Sample	IC_50_ (µg/mL)	
	Acetylcholinesterase	α- glucosidase
SL oil	144 ± 1.04	NA
SL extract	NA	124.6 ± 1.07
Acarbose	--	78.90 ± 1.02
Donepezil	0.00315 ± 1.06	--

### Evaluation of the α-glucosidase and α-amylase inhibitory activities of *Salvia lanigera* extract and essential oil

Alzheimer’s disease (AD) is the most prevalent form of dementia, accounting for 60–80% of all cases. Apparently, AD is one of the main reasons why older people worldwide are becoming less functional in their day-to-day activities. Synaptic dysfunction, neuronal death, behavioural changes and impaired cognitive functions are the main characteristics of AD^45^. Notably, impaired control of blood sugar levels may elevate the risk of developing AD. Hence, AD is currently regarded as type 3 diabetes as impaired glucose metabolism has been shown to be an important regulatory factor in the onset and progression of AD^46^.

Diabetes is a chronic metabolic disease characterized by high levels of blood glucose resulting from defects in the secretion of insulin, its action, or both, which finally leads to serious damage to various organs in the human body. The prevalence of type 2 diabetes (T2D) has risen dramatically in many countries around the world, accounting for 90–95%^47^. Lowering the elevated blood glucose levels is critical for preventing diabetes and associated complications, as it can lead to life-threatening issues such as cerebrovascular and cardiovascular disorders^48^. Carbohydrate molecules are digested to their respective monosaccharides by intestinal enzymes until they are absorbed, causing an increase in blood glucose levels postprandially. *α*-glucosidase is the most important enzyme in carbohydrate digestion^49^. As a result, inhibiting *α*-glucosidase allows for a slower rate of carbohydrate digestion, resulting in lower blood sugar levels and suppression of postprandial hyperglycaemia.

In this study, the in vitro data exhibited that the ethanol extract of *S. lanigera* had potential antidiabetic inhibition activity toward *α*-glucosidase enzyme with an IC_50_ value of 124.6 ± 1.07 µg/mL (Table 4). Conversely, the ethanol extract showed a weak inhibition activity toward *α*-amylase with 18.08 ± 1.42% inhibition, whereas the EO revealed inhibition activity of 27.22 ± 1.90%.

Inhibition of carbohydrate metabolism is a useful therapeutic approach in the management of diabetes and other metabolic disorders. Herein, the results obtained demonstrated that the ethanol extract of *S. lanigera* showed important inhibition effect against α-glucosidase enzyme.

It was observed that phenolic derivatives, particularly flavonoids and phenolic acids, have been attributed to managing the complications of metabolic diseases. These compounds can inhibit digestive enzymes’ activity associated with carbohydrate and lipid metabolism, including α-glucosidase, α-amylase and pancreatic lipase. The *Salvia* species, including *S*. *officinalis*, *S*. *greggii*, and *S*. *elegans* were evaluated for their inhibition potency of enzymes responsible for carbohydrate metabolism. There is a potential correlation between the phenolic content of these plants and the inhibition effect toward α-glucosidase enzyme^50,51^. This possibility could be attributed to caffeic acid and its derivatives. Moreover, a molecular docking screening of polyphenols, such as caffeic acid, daidzein, hesperetin, naringenin, quercetin, and kaempferol toward the α-glucosidase activity suggested significant inhibition of the α-glucosidase enzyme^52^. Besides, a literature survey indicated that rosmarinic acid could efficiently inhibits the α-glucosidase enzyme activity with lower EC_50_ value than acarbose. Importantly, the antidiabetic activity of *Salvia* species is related to the inhibition of α-glucosidase enzyme due to the presence of rosmarinic acid^53,54^. In the current study, marked higher contents of phenolic acid derivatives, including coumaric acid, caffeic acid, gallic acid, chlorogenic acid, ellagic acid, syringic acid, ferulic acid, vanillin, and rosmarinic acid, as well as flavonoids, including kaempferol, naringenin, and hesperetin, were determined. These bioactive molecules characterized in *S. lanigera* support its potential utilization as a significant source of antidiabetic therapies.

### Molecular Docking study

**Fig. 5:**
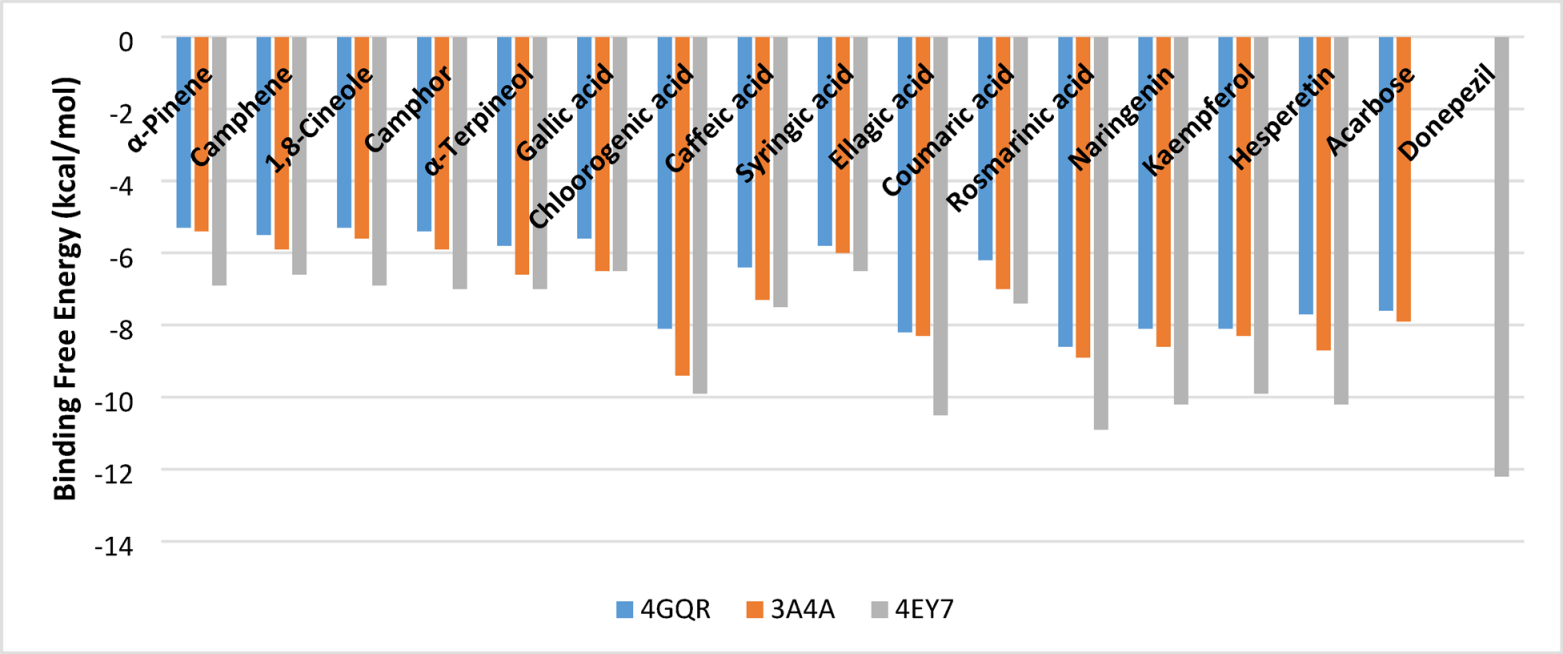
Binding free energy of the major constituents of *S. lanigera* oil and ethanolic extract to α-amylase (4GQR), α-glucosidase (3A4A), and acetylcholinesterase (4EY7).

The study utilized a molecular docking assay to assess the inhibitory effects of major volatiles, phenolic acids, and flavonoids on α-amylase, α-glucosidase, and acetylcholinesterase, complementing in vitro studies. The chosen crystal structures were 4GQR for α-amylase, 3A4A for α-glucosidase, and 4EY7 for acetylcholinesterase^55–57^. Chlorogenic, ellagic, and rosmarinic acids exhibited potent inhibition against the enzymes, with docking scores from − 8.1 to −9.4 kcal/mol, outperforming the control acarbose (−7.6 and − 7.9 kcal/mol) as seen in Fig. 5. Flavonoids followed with scores between − 7.7 and − 8.7 kcal/mol, while monoterpenes from *Salvia lanigera* oil showed weaker binding energies of −5.3 and − 6.6 kcal/mol. The same trend was observed for the docking against acetylcholinesterase, where chlorogenic, ellagic, rosmarinic acids, and flavonoids have the highest comparable binding energies among the examined ligands, ranging from − 9.9 to −10.9 kcal/mol. However, all the ligands examined were lower than the control, donepezil (−12.2 kcal/mol). To our knowledge, nothing has been reported for in-silico studies with *S. lanigera* oil or extract constituents. However, many studies have revealed the interaction through molecular docking for essential oils and botanical extracts with a common trend of higher binding affinities for phenolics compared to terpenes against the same enzymes illustrated in the current study^58–61^.

**Fig. 6:**
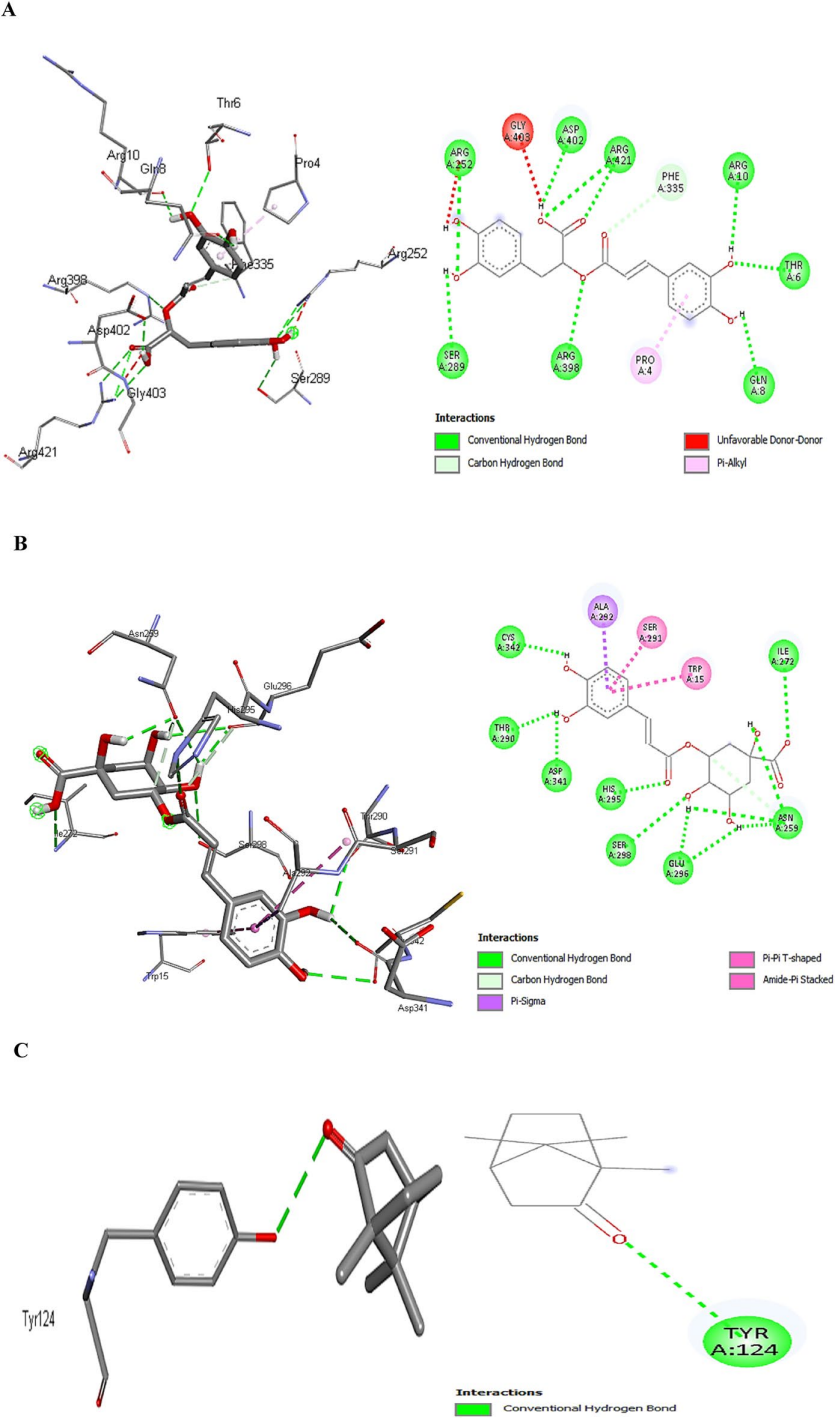
The interactions of rosmarinic acid and 4GQR (**A**), chlorogenic acid and 3A4A (**B**), and camphor and 4EY7 (**C**) (Visualized by Discovery Studio Visualizer ver25.1.0.24284).

Figure 6 revealed the types of binding interactions of 4GQR-rosmarinic acid, 3A4A-chlorogenic acid, and 4EY7-camphor complexes, which showed the highest docking scores among all phytochemicals examined. Eleven conventional hydrogen bonds between the hydroxyl, imino, and amino groups of rosmarinic acid, THR A:6, ARG A:421, ARG A:252, and ARG A:398, as proton donors, and hydroxyl, carbonyl, and carboxylic of rosmarinic acid, ARG A:10, GLN A:8, SER A:289, and ASP A:402 as proton acceptors, were responsible for such higher docking score (Fig. 6A). Surprisingly, the same number of conventional H-bonding observed in 3A4A-chlorogenic complex, also between donors such as amino, imino, and hydroxyl groups of ILE A:272, HIS A:295, SER A:298, and chlorogenic acid, and acceptors as hydroxyl and carbonyl groups of chlorogenic acid, THR A:290, ASP A:341, and CYS A:342 (Fig. 6B). Finally, only one bond observed between the hydroxyl group of TYR A:124 as a donor and the camphor carbonyl group as an acceptor (Fig. 6C).

N–H⋯O interactions were more prevalent than O–H⋯O and N–H⋯N interactions, with neutral and charged hydrogen bonds occurring equally. Proteins primarily acted as hydrogen bond donors, with glycine frequently serving as both an acceptor and donor due to its flexibility. Arginines formed more hydrogen bonds than lysines, likely because of the three nitrogen atoms in arginine’s guanidinium group. Charged O–H⋯O interactions, mainly between alcohols and carboxylic acids, were three times more common than neutral ones, with ligands more often acting as donors. Aspartic acids were the main acceptors for charged bonds, while asparagine, glycine, and glutamine were typical in neutral interactions. Serine was the most common donor^62^.

Carbon-hydrogen bonds could be observed from the methine groups of PHE A:335, HIS A:295, and chlorogenic acid to the carbonyl groups of rosmarinic acid, chlorogenic acid, and ASN A:259 (Figs. 6A and B). Aryl rings are important for hydrophobic interactions in proteins, particularly with amino acids like Trp, which often present their aromatic side chains at binding sites. Aromatic rings’ unique shape and electronic properties allow for favorable interaction geometries, primarily T-shaped edge-to-face and parallel-displaced stacking^63^. Hydrophobic π-π T-shaped interaction was observed between TRP A:15 and the pi-orbitals of chlorogenic acid (Fig. 6B). Similarly, amide.π-stacking interaction was observed in 3A4A-chlorogenic acid complex, where the π-surface of the amide bond (SER A:291 and ALA A:292) stacks against the π-surface of the ligand aromatic ring (Fig. 6B). Finally, hydrophobic π-alkyl interactions could be observed between the π-orbitals of rosmarinic acid and the alkyl group of PRO A:4 (Fig. 6A). The π- σ hydrophobic interaction from the C-H of ALA A:292 to π-orbitals of chlorogenic acid agreed with the ranking of the donor residues reported by Brandl et al.^64^.

Studying molecular docking between major phytochemicals and target enzymes can help reveal interactions in the ligand-receptor complex. However, deviations from in vitro or in vivo results are expected due to factors not considered in in-silico studies. For example, IC_50_ values in in-vivo studies account for absorption, distribution, metabolism, and excretion (ADME), which docking does not. A compound may show a high docking score but fails to reach the target in vivo, resulting in a high IC_50_. Moreover, proteins are flexible, and environments are dynamic, while docking typically uses rigid protein structures and idealized conditions^65^.

Docking may overlook factors like water, ions, or buffer components that affect in vitro binding. Assay conditions (pH, ionic strength) can change enzyme-ligand interactions, leading to deviations from docking predictions. Issues like poor solubility or degradation of phytochemicals can reduce efficacy, and non-specific inhibition from aggregation is not captured in docking. Moreover, in vitro assays may include substrates or cofactors not considered in simulations^66–68^. For example, AChE activity is pH-sensitive, with an optimum around pH 8. Docking simulations typically assume idealized protonation states, while experimental pH affects ligand/protein charge and hydrogen bonding. In vitro assays may involve substrates or co-factors that compete with inhibitors, which docking often overlooks. Additionally, cations or detergents can influence ligand binding stability, and kinetic assays are necessary for irreversible or slow-binding inhibitors, which docking cannot mimic.

In agreement with our findings, Benayad et al.^69^ studied the effect of various solvent extracts and essential oils of Moroccan *Citrus aurantium* (L) peel against α-amylase and α-glucosidase. Ethanol, acetone, and chloroform extracts inhibited the two enzymes by approximately 98%. In contrast, the essential oil was inactive among the examined extracts. There were strong correlations between the phenolics identified and enzyme inhibition, which was verified by molecular docking. In the same line, the best inhibitory activity on acetylcholinesterase was exhibited by the *Thymus algeriensis* oil. On the other hand, *Teucrium polium* oil was more efficient against butyrylcholinesterase, whereas ethanol extracts of the previous plants showed weak or no inhibitory effect, particularly against acetylcholinesterase^69^.

## Conclusion

In summary, according to the results of the HPLC, *Salvia lanigera* growing in Libya was found to contain higher polyphenol and flavonoid components in its polar ethanol extract. Among phenolics and flavonoids, rosmarinic acid, gallic acid, chlorogenic acid, caffeic acid, coumaric acid, kaempferol, naringenin, and hesperitin were dominant. Moreover, this research explored the oil composition of S. *lanigera* and indicated the presence of various hydrocarbons and their oxygenated derivatives. *Salvia lanigera* polar ethanol extract and essential oil showed potent antioxidant activity. Only *S. lanigera* essential oil at a concentration of 500 µg/mL demonstrated inhibition activity toward AChE. Whilst the ethanol extract displayed an inhibition activity toward the α-glucosidase enzyme. The obtained results revealed that *S. lanigera*, which originated from Libya, is a promising source of natural constituents, including polyphenols, flavonoids, and volatile constituents, and possesses beneficial biological properties that can be potentially employed to create novel therapeutic agents for medicinal applications. We found a correlation between IC_50_ values and in-silico results for antidiabetic assays, but not with the anticholinesterase assay. It is well-known that the nature of assays and ligands, reaction environmental conditions, and ADMET may be responsible for the deviation of the in-silico results compared to in-vitro or in-vivo findings. In vivo studies are necessary to confirm the in vitro findings of *S. lanigera* oil and its ethanolic extract. These investigations will provide a clear understanding of the therapeutic potential and efficacy of these compounds in treating diabetes and related conditions, ultimately guiding future drug development efforts. Ultimately guiding future drug development efforts, it is essential to explore the mechanisms of action of *S. lanigera* oil and its extracts.

## Electronic supplementary material

Below is the link to the electronic supplementary material.

Supplementary Material 1

## Data Availability

All data generated or analysed during this study are included in this published article and its supplementary information files.

## Abbreviations

ABTS: (2,2′-azino-bis(3-ethylbenzothiazoline-6-sulfonic acid))
AChE: Acetylcholinesterase enzyme
AD: Alzheimer’s disease
ADMET: Absorption, distribution, metabolism, and excretion
BACE1: Beta-site amyloid precursor protein cleaving enzyme 1
BChE: Butyrylcholinesterase
DPPH: 2,2-diphenyl-1-picrylhydrazyl
DM: Diabetes mellitus
EO: Essential oil
GC–MS: Gas Chromatography–Mass Spectrometry
GSK-3β: Glycogen synthase kinase-3 beta
HPLC–DAD: High-Performance Liquid Chromatography with Diode-Array Detection
RI: Retention indices
LRI: Retention indices according to literature
SL: *Salvia lanigera*
T2D: Type 2 diabetes
TPC: Total phenolic content
TFC: Total flavonoid content
GAE/g: Gallic acid equivalent per gram
