# Supplementary material for: GC-MS and HPLC chemical profile, antioxidant, anti-acetylcholinesterase, and anti-diabetic activities of Libyan Salvia lanigera herb extract and essential oil
Source: Sci Rep. 2025 Aug 29;15:31853. doi: 10.1038/s41598-025-12233-x (PMC12397425; doi:10.1038/s41598-025-12233-x)
Supplement: Supplementary file 1 — Supplementary Material 1 [file 41598_2025_12233_MOESM1_ESM.docx]

**A: 4GQR RMSD=****0.941Å**

The cocrystallized and docked ligands are depicted as sticks in pink and blue, respectively.


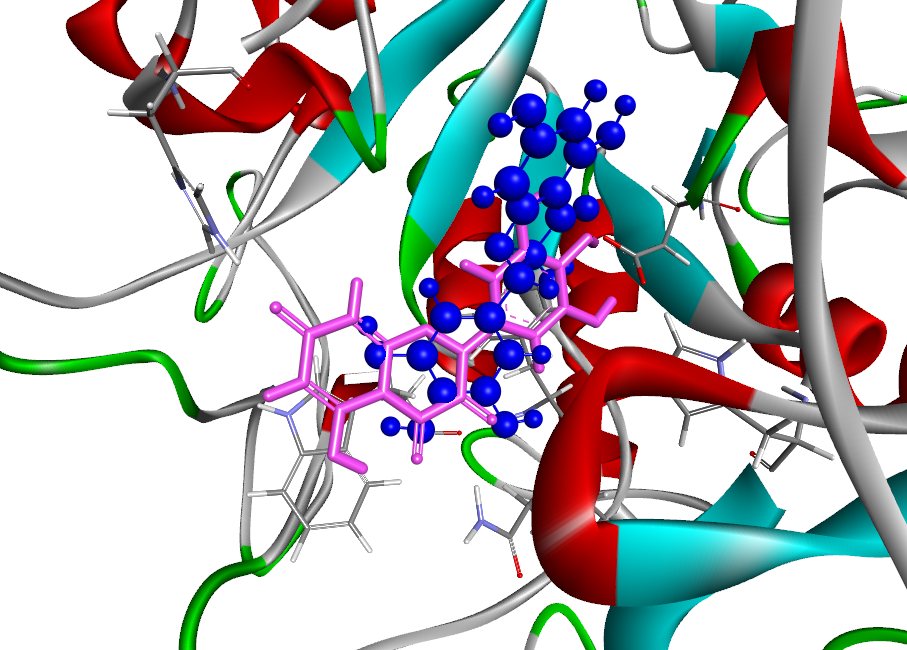


**B: 3A4A** **RMSD=****0.547Å**

The cocrystallized and docked ligands are depicted as sticks in green and red, respectively.


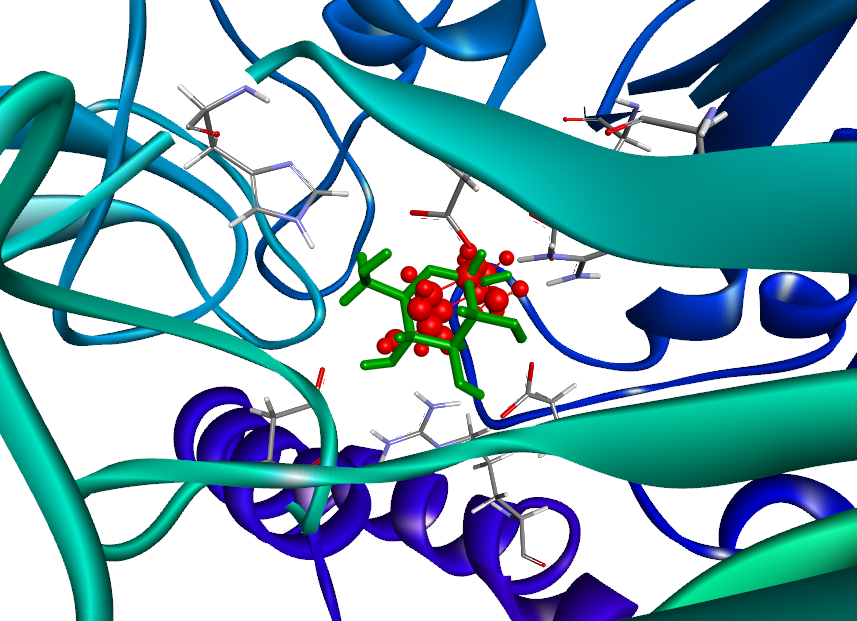


**C: 4EY7 RMSD=0.757Å**

The cocrystallized and docked ligands are depicted as sticks in green and red, respectively.


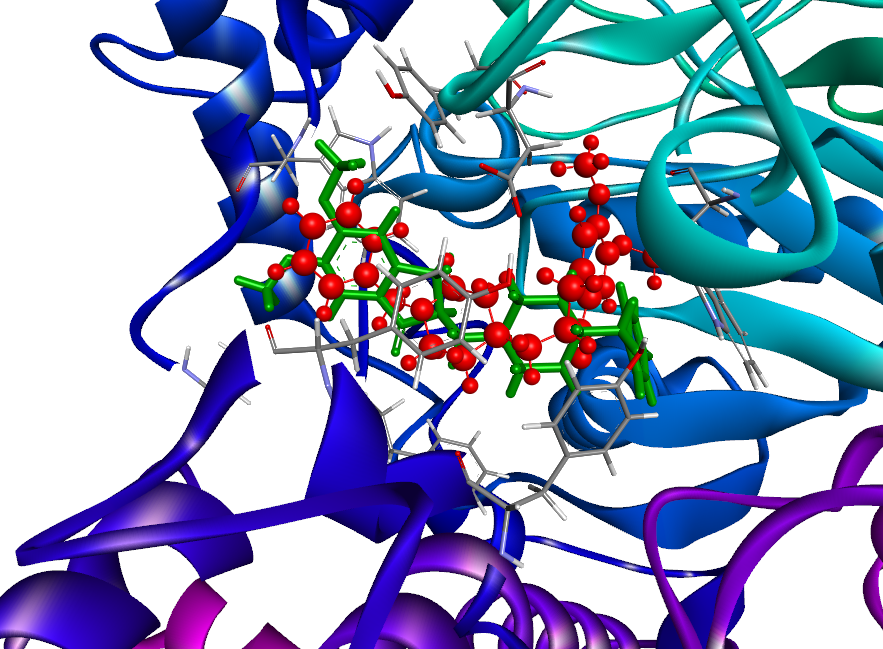


**Figure S1.** The validation of docking performance by AutoDock Vina for proteins and co-crystal ligands (Visualized by Discovery Studio Visualizer ver25.1.0.24284).
